# Mortality hazards after treatment completion of pulmonary tuberculosis in a tertiary hospital in Uganda

**DOI:** 10.1016/j.ijregi.2025.100758

**Published:** 2025-09-10

**Authors:** Felix Bongomin, Martha Namusobya, Ritah Nantale, Daniel S. Ebbs, Charles Batte, Norman van Rhijn, Joseph Baruch Baluku, David W. Denning

**Affiliations:** 1 Manchester Fungal Infection Group, Division of Evolution, Infection and Genomics, Faculty of Biology, Medicine and Health, University of Manchester, Manchester, United Kingdom; 2 Department of Medical Microbiology and Immunology, Faculty of Medicine, Gulu University, Gulu, Uganda; 3 Department of Internal Medicine, Gulu Regional Referral Hospital, Gulu, Uganda; 4 School of Medicine, College of Health Sciences, Makerere University, Kampala, Uganda; 5 Department of Community and Public Health, Faculty of Health Sciences Mbale, Busitema University, Mbale, Uganda; 6 Section of Critical Care Medicine, Department of Paediatrics, Yale University, New Haven, USA; 7 Division of Pulmonology, Kiruddu National Referral Hospital, Kampala, Uganda

**Keywords:** Post-tuberculosis mortality, Chronic pulmonary aspergillosis, Health-related quality of life (HRQoL), TB-HIV coinfection, Resource-limited settings

## Abstract

**Objectives:**

Treated pulmonary tuberculosis (PTB) is associated with long-term complications that contribute to substantial morbidity and mortality. We estimated the incidence and predictors of post-PTB mortality and evaluated whether chronic pulmonary aspergillosis (CPA) independently increases the risk of death.

**Methods:**

Between July 1, 2020 and June 30, 2021, we enrolled 162 individuals with treated drug-susceptible PTB who had persistent respiratory symptoms and screened them for CPA using a symptom checklist, chest x-ray, *Aspergillus* immunoglobulin G-immunoglobulin M point-of-care test, and sputum culture. Between November and December 2024, we followed up all participants via phone calls to determine their vital status. On chest X-ray, PTB was classified as minimal, moderate, and far advanced disease based on involvement of one, two, or more zones, respectively, and coupled to unilateral or bilateral lung disease. Cox proportional hazards regression was used to identify independent predictors of mortality.

**Results:**

Thirty-seven (22.8%) participants were lost to follow-up. The median follow-up duration was 3.8 years (interquartile range 3.6-3.9). Of the 125 participants with vital status, their mean age was 33.5 years (±11.7). At baseline, 46 (36.8%) had far advanced PTB, 64 (51.2%) had pulmonary fibrosis, 15 (12.0%) had a history of previous TB, and 34 (27.2%) were living with HIV. Coinfection with PTB and CPA was identified in 31 participants at baseline (24.8%). The median St. George’s respiratory questionnaire score was 50.9 (interquartile range 40.9-63.3), and 32.0% (n = 40) had scores above 60, indicating poor health-related quality of life. Overall mortality was 8.8% (95% confidence interval [CI] 4.4-15.2%), with a mortality rate of 24.3 deaths per 1000 person-years of follow-up. Mortality rates were comparable among participants with and without CPA-PTB coinfection. Independent predictors of mortality included a St. George’s respiratory questionnaire score >60 (adjusted hazard ratio = 2.01; 95% CI 1.49-2.72; *P* <0.001) and HIV infection (adjusted hazard ratio = 3.04; 95% CI 1.46-6.34; *P* = 0.029).

**Conclusions:**

Post-PTB mortality remains high, with poor health-related quality of life and HIV co-infection emerging as significant independent predictors of death. Integrating long-term follow-up, respiratory rehabilitation, and fungal diagnostics into post-TB care pathways is essential to improve outcomes and reduce preventable mortality.

## Highlights

• High post-tuberculosis (TB) mortality (8.8%) linked to poor quality of life and HIV.
• Chronic pulmonary aspergillosis coinfection (24.8%) did not significantly raise mortality risk.
• Far advanced pulmonary TB (36.8%) and fibrosis (51.2%) were common at baseline.
• St. George’s respiratory questionnaire score >60 and HIV tripled mortality risk (adjusted hazard ratio 2.01 and 3.04).
• Post-TB care needs rehab, fungal screening, and long-term follow-up.

## Introduction

Tuberculosis (TB) remains a leading global public health challenge, with an estimated 10.8 million new cases and 1.25 million deaths reported in 2023, including 161,000 among people living with HIV [1]. Despite significant advancements in TB diagnosis and treatment, post-TB mortality remains alarmingly high, with survivors facing a three-fold increased risk of death compared with the general population [2,3]. Pulmonary TB (PTB), the most common form of the disease, is associated with long-term respiratory complications such as pulmonary fibrosis, bronchiectasis, and chronic obstructive pulmonary disease, which contribute to morbidity and mortality even after successful treatment [[4], [5], [6]].

The burden of post-TB sequelae is particularly pronounced in sub-Saharan Africa, where high rates of HIV coinfection and limited access to specialized care exacerbate poor outcomes [7]. HIV infection is a well-established risk factor for TB mortality, but its role in post-treatment mortality among PTB survivors remains understudied [3,[7], [8], [9]]. In addition, chronic pulmonary aspergillosis (CPA), a debilitating fungal infection often complicating PTB, further increases the risk of death, yet its contribution to post-TB mortality is poorly characterized in high-burden settings [10,11].

Emerging evidence highlights the critical role of health-related quality of life (HRQoL) in predicting post-TB mortality and morbidity [12,13]. The St. George’s respiratory questionnaire (SGRQ), a validated tool for assessing respiratory health, has been linked to increased mortality in chronic lung diseases, including post-TB lung disease [14,15]. However, data on the association between HRQoL and post-TB mortality in resource-limited settings are scarce.

Uganda, a high TB/HIV burden country, faces unique challenges in post-TB care, including fragmented follow-up systems, few centers doing spirometry or computed tomography scans, patients having to pay for their diagnostics [16,17], and limited integration of fungal diagnostics [18]. Previous cohort studies have reported post-PTB mortality between 10% and 20% [9,19,20]. Understanding the predictors of post-PTB mortality in this context is essential for developing targeted interventions. We determined the incidence and predictors of mortality among PTB survivors in Uganda, with a focus on the roles of HIV coinfection, CPA, and HRQoL.

## Methods

### Study design and setting

This prospective cohort study was conducted at the National Tuberculosis Control Center of Mulago National Referral Hospital (MNRH) in Kampala, Uganda. The study period for enrollment spanned from July 1, 2020 to June 30, 2021 [21,22], with follow-up extending through December 2024. MNRH serves as Uganda's largest TB treatment facility, managing approximately 1500 patients with TB annually (Ministry of Health Uganda, 2020). The hospital employs a mixed model of care, incorporating both inpatient management for severely ill patients and outpatient community-based, directly observed therapy for stable cases. However, there is no structured post-TB care program across the country.

### Study population and eligibility criteria

The study enrolled adults aged 18 years or older with microbiologically confirmed drug-sensitive PTB, diagnosed using the GeneXpert MTB/RIF assay. Eligible participants exhibited persistent pulmonary or systemic symptoms of cough, progressive shortness of breath, sputum production, and/or hemoptysis, despite completion of at least 2 months of standard anti-TB therapy (initiation phase). Exclusion criteria included extrapulmonary TB, critical illness precluding informed consent, pregnancy, and the use of second-line anti-TB regimens, which indicated potential drug resistance.

### Data collection procedures

#### Baseline assessments (2020-2021)

Baseline data collection encompassed sociodemographic, clinical, and laboratory measures. Sociodemographic variables included age, sex, marital status, education level, income, and history of smoking or alcohol use, assessed using Yes/No questions. Clinical characteristics were documented, including TB disease severity, classified as minimal, moderate, and far advanced disease based on involvement of one, two, or more zones, respectively, and coupled to unilateral or bilateral lung disease [23]. Comorbidities such as HIV status, confirmed through national testing protocols, and prior TB episodes were recorded. Respiratory symptoms, including persistent cough, hemoptysis, dyspnea, chest pain, and night sweats, were systematically assessed.

Participants were screened for CPA using a symptom checklist, chest X-ray findings (e.g., cavities, fibrosis, fungal balls), *Aspergillus* immunoglobulin (Ig)G-IgM serology (LDBio IgG-IgM POCT and Bordier *Aspergillus* IgG ELISA), and sputum culture for *Aspergillus*, performed at the Makerere University Infectious Diseases Institute Translational Laboratory. A participant was classified as having CPA if they had persistent respiratory symptoms (at least a cough or hemoptysis lasting for 3 months or more), suggestive chest X-ray findings (cavities, pericavitary infiltrates, a fungal ball [intracavitary content], pericavitary fibrosis, or pleural thickening), and evidence of *Aspergillu*s infection (a positive HVS culture and/or *Aspergillus* IgG/IgM ICT) consistent with the Global Fungal Infection Forum II diagnostic criteria for CPA in resource-limited settings [24]. HRQoL was evaluated using the SGRQ, with scores categorized into quartiles (<32, 32-46, 46-60, >60) as previously validated [25]. Higher scores indicated poorer HRQoL [26].

#### Follow-up procedures (2024)

During the follow-up phase in November-December 2024, vital status was determined for all participants through structured phone interviews with the participants or their relatives. Mortality rates were calculated as the number of deaths per 1000 person-years of follow-up.

### Statistical analysis

Data analysis was performed using Stata 18.0 (StataCorp, College Station, TX). Descriptive statistics summarized continuous variables as means with standard deviations (SD) or medians with interquartile ranges (IQR), as appropriate. Categorical variables are presented as frequencies and percentages. Survival analysis included Kaplan-Meier curves to estimate survival probabilities stratified by HIV and CPA status. Cox proportional hazards regression models identified predictors of mortality. Variables with a *P* <0.2 in bivariable analysis were included in the final multivariable model. Adjusted hazard ratios (aHR) with 95% confidence intervals (CI) were reported to quantify associations. Participants lost to follow-up (n = 37, 22.8%) were excluded from mortality rate calculations.

### Ethical considerations

For the baseline study, the protocol received approval from the Makerere University School of Biomedical Sciences Research Ethics Committee (SBS-795) and the Uganda National Council for Science and Technology (HS739ES). Written informed consent was obtained from all participants prior to enrollment, ensuring adherence to ethical standards and respect for participant autonomy. For the follow-up phase, additional approvals were provided by the Gulu University Research Ethics Committee (#GUREC-2023-717), the University of Manchester University Research Ethics Committee 2 (Ref: 2024-20473-37733), and the Uganda National Council for Science and Technology (HS5799ES).

## Results

### Participant selection and verbal autopsy

**Figure 1:**
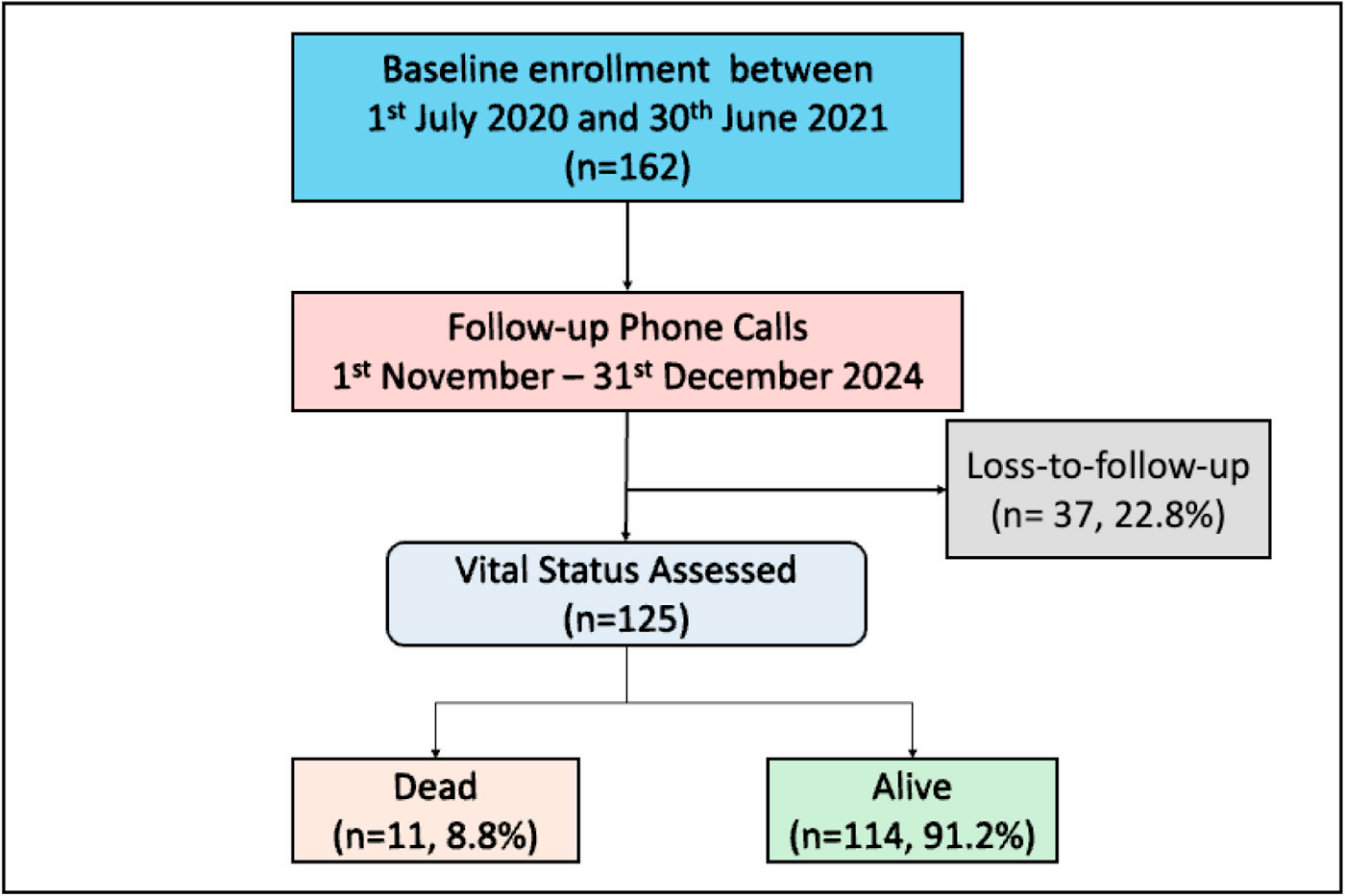
Participant selection and outcome.

Figure 1 shows the enrollment and follow-up schedule. Of the 11 individuals who died, one death was due to a road traffic accident, one to lung cancer, and nine were reported to have had a progressive PTB disease despite treatment completion. Eight of the nine individuals who died from progressive disease died while in hospital for post-TB complications.

### Sociodemographic characteristics

Of the 162 participants, 125 were analyzed. The mean age (SD) of all the participants was 33.5 ± 11.7 years. More than half (56.8%, n = 71) were single and 53.2% (n = 66) had attained secondary education level. The majority (94.4%, n = 118) had no history of smoking and 28.8% (n = 36) had a history of alcohol use (Table 1).

**Table 1:** Baseline sociodemographic characteristics.

Characteristic (n = 125)	Frequency (%)
Sex	
Female	52 (41.6%)
Male	73 (58.4%)
Age in years, mean ± SD	33.5 ± 11.7
18-24	29 (23.4%)
25-34	48 (38.7%)
>35	47 (37.9%)
Marital status	
Married	54 (43.2%)
Single	71 (56.8%)
Education level	
None/Primary	44 (35.5%)
Secondary	66 (53.2%)
Tertiary	14 (11.3%)
Work status	
No	76 (60.8%)
Yes	49 (39.2%)
Monthly income (n = 49), median (interquartile range)	UGX 300,000 (200,000-350,000)
Smoking	
No	118 (94.4%)
Yes	7 (5.6%)
Alcohol use	
No	89 (71.2%)
Yes	36 (28.8%)

### Clinical characteristics of the participants

Most of the participants, 36.8% (n = 46) had far advanced TB disease. More than half, 51.2% (n = 64) had fibrosis in the lung based on X-ray findings at baseline. Regarding presenting complaints, all participants had a persistent cough, 92.0% (n = 115) had chest pain, 66.4% (n = 83) had night sweats, 48.8% (n = 61) had weight loss, 43.2% (n = 61) had fatigue, and 40.8% (n = 51) had shortness of breath. Twelve percent (n = 15) had prior TB and 27.2% (n = 34) were HIV positive. Nearly a quarter, 24.8% (n = 31) had TB and CPA. The median (IQR) SGRQ score was 50.9 (40.9-63.3) and 32.0% (n = 40) had a score >60 (Table 2).

**Table 2:** Clinical characteristics of the participants after 2 months of anti-tuberculosis therapy.

Characteristic (n = 125)	Frequency (%)
Disease extent	
Far advanced disease	46 (36.8%)
Minimal disease	18 (14.4%)
Moderately advanced disease	41 (32.8%)
Normal	20 (16.0%)
Baseline X-ray findings (2 months into TB treatment)	
Fibrosis	64 (51.2%)
Cavity	40 (32.0%)
Pleural effusion	18 (14.4%)
Fungal ball	5 (4.0%)
Presenting complaints	
Persistent cough	125 (100.0%)
Hemoptysis	19 (15.2%)
Shortness of breath	51 (40.8%)
Weight loss	61 (48.8%)
Fatigue	54 (43.2%)
Fevers	24 (19.2%)
Appetite loss	45 (36.0%)
Night sweats	83 (66.4%)
Chest pain	115 (92.0%)
Wheezing	14 (11.2%)
Had prior TB	15 (12.0%)
HIV status	
Negative	91 (72.8%)
Positive	34 (27.2%)
Aspergillosis status	
TB alone	94 (75.2%)
TB + chronic pulmonary aspergillosis	31 (24.8%)
SGRQ score, median (IQR)	50.9 (40.9-63.3)
Symptoms score, median (IQR)	52.7 (42.9-66.0)
Activity score, median (IQR)	60.3 (47.7-72.8)
Impact score, median (IQR)	44.3 (31.2-56.1)
SGRQ Quartiles	
<32	14 (11.2%)
32 to 46	34 (27.2%)
46 to 60	37 (29.6%)
>60	40 (32.0%)

IQR, interquartile range; SGRQ, St. George’s respiratory questionnaire; TB, tuberculosis.

### Mortality among people who have completed treatment for pulmonary tuberculosis in Uganda

Of the 125 participants enrolled, 8.8% (95% CI 4.4-15.2%) died. The mortality rate was 24.3 per 1000 person-years. The median person time of follow-up was 3.8 years (IQR 3.6-3.9).

### Predictors of mortality among people who have completed treatment for pulmonary tuberculosis in Uganda

**Figure 2:**
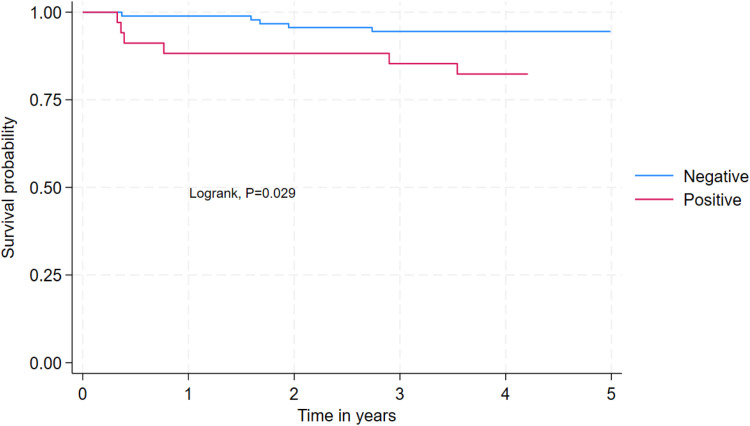
Survival among participants with HIV compared with those without HIV.

**Figure 3:**
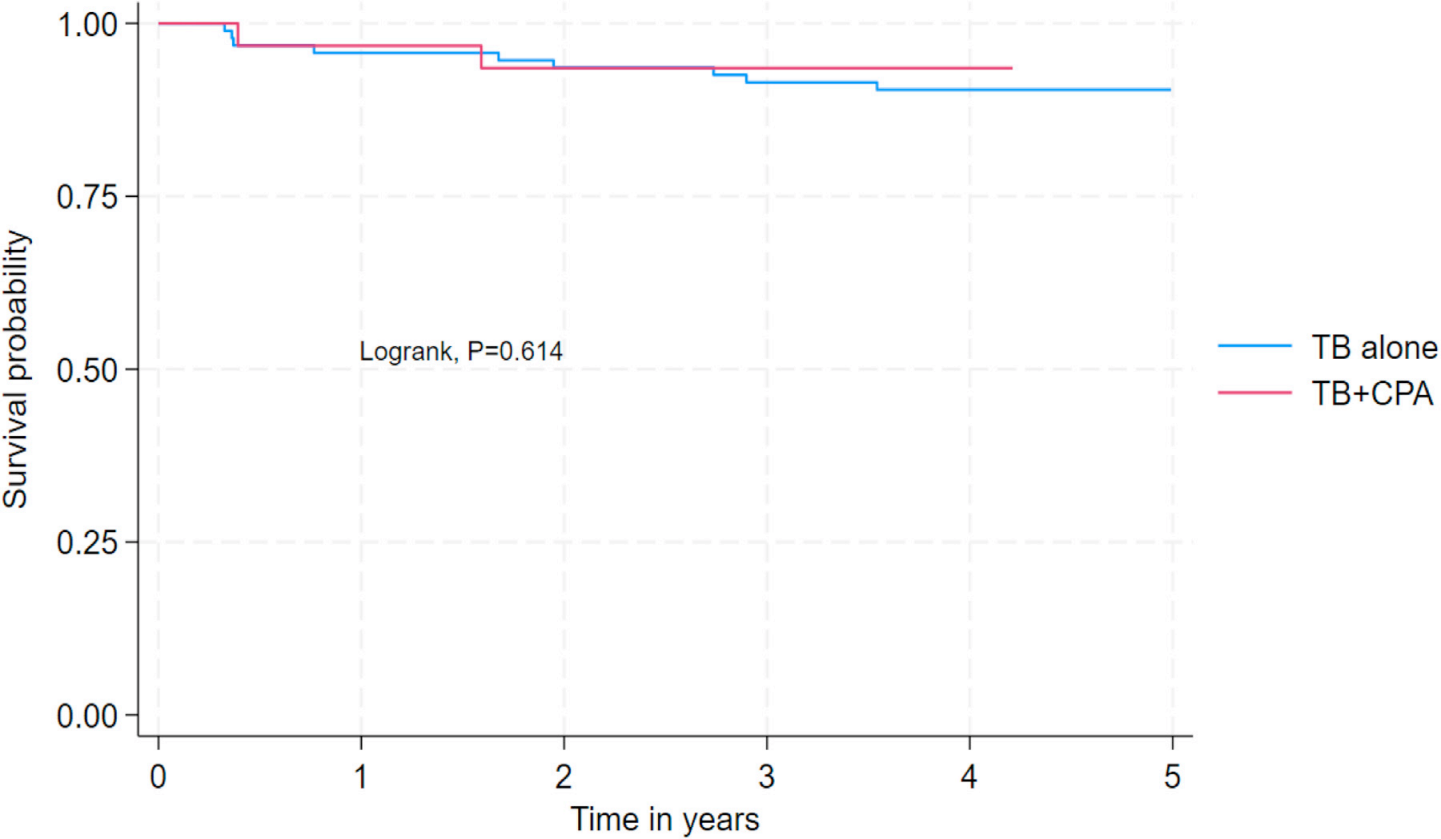
Survival among participants with tuberculosis alone compared with those with TB and co-infection.

CPA, chronic pulmonary aspergillosis; TB, tuberculosis.

Participants with an SGRQ score >60 had a two-fold higher hazard of mortality compared with those with an SGRQ score <32 (aHR = 2.01, 95% CI 1.49-2.72, *P* <0.001). Participants living with HIV had a three-fold higher hazard of mortality compared with those who are living without HIV (aHR = 3.04, 95% CI 1.46-6.34, *P* = 0.029) (Figure 2) (Table 3). Mortality rates were comparable among participants with CPA-PTB coinfection (2/31 [6.5%] vs 9/94 [9.6%], log-rank = 0.614) (Figure 3). Both deceased cases of CPA-PTB coinfection had *Aspergillus niger* isolated from their sputum and far advanced disease with bilateral fibrosis and pleural effusion.

**Table 3:** Predictors of mortality among people who have completed treatment for pulmonary tuberculosis in Uganda.

Variable	Alive, n (%) n = 114	Dead, n (%) n = 11	Crude HR	95% CI	*P*-value	Adjusted HR	95% CI	*P*-value
Sex								
Female	48 (42.1%)	4 (36.4%)	1					
Male	66 (57.9%)	7 (63.6%)	1.24	(0.36-4.25)	0.728			
Marital status								
Married	49 (43.0%)	5 (45.5%)	1					
Single	65 (57.0%)	6 (54.5%)	0.92	(0.28-3.01)	0.889			
Education level								
None/Primary	37 (32.7%)	7 (63.6%)	1			1		
Secondary	63 (55.8%)	3 (27.3%)	0.27	(0.07-1.05)	0.058	0.47	(0.10-2.21)	0.342
Tertiary	13 (11.5%)	1 (9.1%)	0.43	(0.05-3.52)	0.434	1.41	(0.20-10.14)	0.733
Smoking								
No	109 (95.6%)	9 (81.8%)	1			1		
Yes	5 (4.4%)	2 (18.2%)	4.51	(0.97-20.91)	0.054	4.06	(0.15-110.32)	0.406
Alcoholism								
No	82 (71.9%)	7 (63.6%)	1					
Yes	32 (28.1%)	4 (36.4%)	1.46	(0.43-4.98)	0.547			
Work status								
No	69 (60.5%)	7 (63.6%)	1					
Yes	45 (39.5%)	4 (36.4%)	0.85	(0.25-2.92)	0.801			
SGRQ score								
<32	12 (10.5%)	2 (18.2%)	1			1		
32 to 46	33 (28.9%)	1 (9.1%)	0.2	(0.02-2.24)	0.193	0.3	(0.05-1.67)	0.168
46 to 60	35 (30.7%)	2 (18.2%)	0.38	(0.05-2.69)	0.333	0.64	(0.09-4.82)	0.665
>60	34 (29.8%)	6 (54.5%)	1.12	(0.23-5.54)	0.892	2.01	(1.49-2.72)	<0.001
HIV status								
Negative	86 (75.4%)	5 (45.5%)	1			1		
Positive	28 (24.6%)	6 (54.5%)	3.44	(1.05-11.27)	0.041	3.04	(1.46-6.34)	0.029
Aspergillosis status								
TB alone	85 (74.6%)	9 (81.8%)	1					
TB + chronic pulmonary aspergillosis	29 (25.4%)	2 (18.2%)	0.68	(0.15-3.13)	0.616			

CI, confidence interval; HR, hazard ratio; TB, tuberculosis.

## Discussion

This prospective cohort study evaluated mortality and its predictors among Ugandan adults who completed treatment for PTB, with a focus on the roles of HRQoL, HIV coinfection, and CPA. Our findings reveal an 8.8% mortality rate (24.3 deaths per 1000 person-years), with poor HRQoL (SGRQ >60) and HIV infection emerging as independent predictors of death. These results align with global evidence that post-TB mortality remains unacceptably high, particularly in sub-Saharan Africa, where health systems face challenges in long-term TB survivor care [2,27]. Our findings are consistent with a retrospective cohort study by Baluku et al. conducted in rural central Uganda, which reported a mortality rate of 11.4%, with hospitalization and unemployment at TB treatment initiation as significant predictors of mortality [19]. Another study by Lumu et al., among people with HIV attending a specialist HIV clinic in Uganda, all-cause mortality was 15% and was associated with advanced HIV disease and retreatment [20]. Another prospective cohort study by Kirenga et al. found an 18% mortality rate in the post-TB treatment period, with mortality rate (per 100 person-years at risk) being significantly higher for people with HIV (41 deaths, rate 46.9 per 100 person-years at risk) than for the HIV negative individuals (11 deaths, rate 19.7 per 100 person-years at risk) [9].

Participants with SGRQ scores >60 had a two-fold higher mortality risk, consistent with studies linking poor HRQoL to post-TB lung damage and premature death [13]. The SGRQ captures multidimensional impairment: symptoms, activity limitation, and psychosocial impact, making it a robust prognostic tool. Our findings support its integration into post-TB monitoring to identify high-risk patients needing rehabilitation [6].

HIV-positive participants had three-fold higher mortality, corroborating evidence that HIV exacerbates post-TB mortality due to immune dysfunction and opportunistic infections [7,28]. Despite Uganda’s scale-up of antiretroviral therapy (ART), TB/HIV co-infected individuals remain vulnerable to late complications, including cardiometabolic derangement [29], highlighting the need for enhanced ART adherence support and cardiovascular disease screening [30].

CPA was identified in 24.8% of participants in our study, yet it did not emerge as an independent predictor of mortality. Mortality in those with CPA-PTB coinfection was a low as 6.5%. This is comparable to our earlier systematic review of CPA in Africa, which showed an overall 6.5% all-cause mortality [31]. In India, under a trial setting, of 164 individuals with PTB associated CPA, 10 (6.1%) died over 12-month period. In our recent systematic review, overall, 1-year mortality in CPA complicating TB was found to be 17% and 5-year mortality in those with post-TB was 27% [11]. Several factors may explain this discrepancy. First, we were only able to follow up 77.2% of patients, and it is possible (even likely) that some of the patients lost to follow had died, underestimating all deaths, and possibly CPA deaths. Second, limited access to advanced diagnostic tools such as CT imaging and *Aspergillus* polymerase chain reaction in Uganda, and a diagnostic sensitivity of *Aspergillus* IgG/IgM of 68-92% for CPA, may have led to underdiagnosis of early CPA cases. Third, the high prevalence of competing risks, including HIV coinfection and advanced lung fibrosis, may have overshadowed CPA's contribution to mortality in this cohort. Finally, the follow-up duration of 3.8 years may have been insufficient to capture the latent impact of CPA. However, CPA-related mortality decreases with time, especially with antifungal therapy and surgical interventions [11]. In our cohort, less than 10% of CPA cases received itraconazole, and by the end of TB therapy, those with CPA-PTB coinfection had worse HRQoL compared with those with PTB only [21].

### Strengths and limitations

Our study has several notable strengths. The prospective design, with a median follow-up of 3.8 years, minimized recall bias and provided robust longitudinal data. The multidimensional assessment of mortality predictors, encompassing clinical, radiological, and HRQoL measures, allowed for a comprehensive analysis. In addition, the real-world setting of a high-burden TB/HIV context enhances the generalizability of our findings to similar resource-limited settings. However, the study also has limitations. A loss to follow-up rate of 22.8% may have led to an underestimation of mortality. However, this loss to follow-up rate is typical among TB survivors in Uganda [9,19]. Diagnostic constraints for CPA, particularly the reliance on serology and chest X-rays rather than more sensitive methods like computed tomography or bronchoalveolar lavage, could have resulted in missed cases. Furthermore, residual confounding from unmeasured factors, such as socioeconomic status and ART adherence, may have influenced the observed outcomes.

### Implications of study findings

The findings of this study emphasize the need for integrated post-TB care strategies. Routine screening of TB survivors for HRQoL impairment using tools like the SGRQ and for CPA through symptom checklists and *Aspergillus* serology could help identify high-risk individuals. Prioritizing respiratory rehabilitation for patients with SGRQ scores >60, as recommended by the international post-tuberculosis symposium [27], may improve outcomes.

For HIV-positive individuals, strengthening ART adherence programs and managing comorbidities such as diabetes and hypertension are critical to reducing mortality. In addition, scaling up affordable *Aspergillus* diagnostics, such as lateral flow assays, in TB clinics could enhance early CPA detection and treatment [32].

Future research should focus on longer-term cohort studies to evaluate the delayed mortality effects of CPA, as our study may have been underpowered to detect these associations. Clinical trials investigating the efficacy of antifungals, such as newer generations of triazoles and or intravenous polyenes or echinocandins, in CPA-PTB coinfection are also warranted. Finally, cost-effectiveness analyses of post-TB care models in resource-limited settings could inform policy decisions and optimize resource allocation.

## Conclusion

This study highlights poor HRQoL and HIV as critical drivers of post-TB mortality in Uganda, while the role of CPA in post-PTB mortality warrants further investigation. Implementing integrated, patient-centered follow-up, incorporating HRQoL assessment, HIV care, and fungal diagnostics, could mitigate preventable deaths among TB survivors. Policymakers must prioritize post-TB morbidity as a key component of TB elimination strategies. Although CPA did not independently predict mortality in our cohort, its high prevalence highlights the need for improved diagnostic and management strategies. Addressing the broader determinants of post-TB mortality, including HRQoL and HIV coinfection, through integrated care models remains a priority for reducing preventable deaths among TB survivors.

## Declaration of competing interest

The author have no competing interests to declare.
